# Apoptosis Induced by the UV Filter Benzophenone-3 in Mouse Neuronal Cells Is Mediated via Attenuation of Erα/Pparγ and Stimulation of Erβ/Gpr30 Signaling

**DOI:** 10.1007/s12035-017-0480-z

**Published:** 2017-03-29

**Authors:** A. Wnuk, J. Rzemieniec, W. Lasoń, W. Krzeptowski, M. Kajta

**Affiliations:** 1 Department of Experimental Neuroendocrinology, Institute of Pharmacology, Polish Academy of Sciences, Smetna Street 12, 31-343 Krakow, Poland; 2 Department of Cell Biology and Imaging, Institute of Zoology, Jagiellonian University, Gronostajowa Street 9, 30-387 Krakow, Poland

**Keywords:** Benzophenone-3, BP-3, Estrogen receptors, Peroxisome proliferator-activated receptor gamma, Primary neuronal cell cultures

## Abstract

Although benzophenone-3 (BP-3) has frequently been reported to play a role in endocrine disruption, there is insufficient data regarding the impact of BP-3 on the nervous system, including its possible adverse effects on the developing brain. Our study demonstrated that BP-3 caused neurotoxicity and activated apoptosis via an intrinsic pathway involving the loss of mitochondrial membrane potential and the activation of caspases-9 and -3 and kinases p38/MAPK and Gsk3β. These biochemical alterations were accompanied by ROS production, increased apoptotic body formation and impaired cell survival, and by an upregulation of the genes involved in apoptosis. The BP-3-induced effects were tissue-specific and age-dependent with the most pronounced effects observed in neocortical cells at 7 days in vitro. BP-3 changed the messenger RNA (mRNA) expression levels of *Erα*, *Erβ*, *Gpr30*, and *Pparγ* in a time-dependent manner. At 3 h of exposure, BP-3 downregulated estrogen receptor mRNAs but upregulated *Pparγ* mRNA*.* After prolonged exposures, BP-3 downregulated the receptor mRNAs except for *Erβ* mRNA that was upregulated. The BP-3-induced patterns of mRNA expression measured at 6 and 24 h of exposure reflected alterations in the protein levels of the receptors and paralleled their immunofluorescent labeling. Erα and Pparγ agonists diminished, but Erβ and Gpr30 agonists stimulated the BP-3-induced apoptotic and neurotoxic effects. Receptor antagonists caused the opposite effects, except for ICI 182,780. This is in line with a substantial reduction in the effects of BP-3 in cells with siRNA-silenced Erβ/Gpr30 and the maintenance of BP-3 effects in Erα- and Pparγ siRNA-transfected cells. We showed for the first time that BP-3-affected mRNA and protein expression levels of Erα, Erβ, Gpr30, and Pparγ, paralleled BP-3-induced apoptosis and neurotoxicity. Therefore, we suggest that BP-3-evoked apoptosis of neuronal cells is mediated via attenuation of Erα/Pparγ and stimulation of Erβ/Gpr30 signaling.

## Introduction

The toxicity of ultraviolet light (UV) filters used in sunscreens and cosmetics to attenuate the negative effects of harmful UV radiation on skin and hair has become a concern. More than approximately 10,000 tons of UV filters are produced annually for the global market [1]. The variety of such compounds and the percentage of different filters added to industrial products are expanding with the increasing demand to prevent UV radiation-induced cell damage. Organic chemicals that absorb UVA (400–315 nm) or UVB (315–280 nm) radiation are added at concentrations of up to 10% to sunscreen products for skin protection. Due to public anxiety regarding skin damage caused by UV light, the use of UV screens is increasing, even though the benefit with respect to the prevention of melanoma remains controversial. In Europe, 28 chemical substances that function as UV filters are approved for use in industry, but the European Commission has reported many of them as hormonally active substances or endocrine-disrupting chemicals (EDCs). Chemical filters are almost always used in the mixtures but none of them are used individually in cosmetic in acceptable concentrations and does not provide adequate protection. These compounds are approved as harmless when used in small doses, but it should be noted that daily use involves at least several different cosmetics.

Benzophenone-3 (2-hydroxy-4-methoxybenzophenone, oxybenzone, 2OH-4 MeO-BP or BP-3) is a commonly used sunscreen agent, absorbing UVB and UVA radiation. The industrial use of BP-3 has increased over the past decade [2]. BP-3 is utilized as a flavor ingredient; a fragrance enhancer; a perfume fixative, an additive for plastics, coatings, and adhesive formulations; an ultraviolet curing agent in sunglasses; and as an agent to prevent UV light from damaging scents and colors in perfumes and soaps. It can be added to plastic packaging as a UV blocker, which allows manufacturers to package their products in clear glass or plastic rather than in dark packaging. Additionally, BP-3 has been approved by the US Food and Drug Administration as an indirect food additive [3]. Population studies have demonstrated that it penetrates through the skin and is excreted in the urine. BP-3 was found in >95% of urine samples collected from the US general population and in 100% of an investigated group of Danish children at concentrations ranging from 0.4 to 21,700 ng/ml [4–8]. In some cases, as much as 10% of the applied dermal dose was absorbed into systemic circulation [9]. BP-3 was found in 83% of blood samples from investigated adults [10].

The majority of reports have concentrated on the negative effects of BP-3 on the reproductive system. Epidemiological studies have provided evidence of a strong correlation between BP-3 exposure and increased ratios of diagnosed endometriosis [11]. However, the most disturbing finding is the fact that breastfed babies are exposed to BP-3, as this UV filter was detected in human milk samples [12]. In addition to mother milk, other ways of exposures to BP-3 are possible. BP-3 was found to cross blood-brain barrier after being applied in gavages [13]. It has been demonstrated that bisphenol A (BPA), which chemical structure is very similar to BP-3, easily crosses the blood–placental barrier [14, 15]. Therefore, fetal brain could be exposed to a much higher concentration of BP-3 than the concentration that was detected in human milk fat by Schlumpf et al. [12]. The high concentrations of BP-3 that have been detected in mother’s urine may reduce birth weight in girls and increase birth weight and head circumference in boys [6, 16]. It has been postulated that the BP-3 concentration in urine may reach 16.7 μg/l and it may reflect less than 1% of the concentration of BP-3 to which healthy volunteers were exposed [17, 18]. Furthermore, a strong association between maternal exposure to BP-3 and the onset of Hirschsprung’s disease in offspring has been shown [19]. In addition to the above-mentioned studies, there is insufficient data on the impact of BP-3 on the nervous system, including its possible adverse effects on the developing brain. The only relevant report showed apoptotic and neurotoxic effects of BP-3 on SH-SY5Y neuroblastoma cells [20].

BP-3 has frequently been reported to play a role in endocrine disruption [21–24]. This disruption can occur by altering normal hormone levels, inhibiting or stimulating the production and metabolism of hormones, or changing the way hormones travel through the body, thus affecting the functions that these hormones control. EDCs have been postulated to exert their actions through nuclear hormone receptors, including estrogen receptors and peroxisome proliferator-activated receptor gamma (PPARγ), either by activating or by suppressing them. Although BP-3 has been recognized as a ligand for nuclear estrogen receptors (ERs), there is no data regarding its interactions with newly identified membrane ER, G protein-coupled receptor 30 (GPR30), and the nuclear receptor PPARγ, which is known to interact with ERs. Identifying chemicals that display hormonal activity is now a major research focus because they could disrupt brain development and cause abnormalities observed during ontogeny or at the onset of neurodegenerative diseases.

Primarily, ERs mediate the actions of endogenous estrogen hormones, which influence a wide variety of developmental and functional aspects in the mammalian central nervous system (CNS). This includes the survival of neurons, the growth and plasticity of neurites, their synaptic connections and transmission, and motor skills and higher cognitive functions [25]. Experimental and epidemiologic studies have provided information that estrogens protect against Alzheimer’s disease (AD) in postmenopausal women and positively affect the symptoms of Parkinson’s disease and tardive dyskinesia [26]. The idea of a neuroprotective function of estrogen is supported by observations that females are less vulnerable to acute insults associated with cerebral ischemia, neurotrauma, and hypoxia and that estradiol or estrogen-like compounds protect against stroke [27, 28]. Surprisingly, EDCs interference with nuclear receptors implicated in metabolism has remained scarcely studied. Peroxisome proliferator-activated receptors are ligand-activated transcription factors that regulate genes important in cell differentiation and various metabolic processes, especially lipid and glucose homeostasis [29]. These receptors are important for neuronal development and brain function. Agonists of PPARγ have been used to reduce neurodegenerative changes in mouse models of neurodegenerative diseases [30] and have also shown benefits in experimental models of stroke and ischemia [31]. Concomitantly, studies of PPARγ activation showed effects against oxidative stress, mitochondrial dysfunction, and apoptosis in several cell models that resemble AD, Huntington’s disease (HD), amyotrophic lateral sclerosis (ALS), and spinal cord injuries (SCI) [32]. A signal cross-talk between nuclear ERs and the PPARγ has also been shown [33]. However, there is no data on the interactions of BP-3 with these receptors in mammalian neurons.

Recently, we showed that the pesticide dichlorodiphenyltrichloroethane (DDT) impaired Gpr30-mediated intracellular signaling in mouse neurons [34]. We also demonstrated an involvement of the retinoid X receptor (Rxr), which is an obligatory heterodimer partner of Pparγ, in the neurotoxic and apoptotic actions of nonylphenol and dichlorodiphenyldichloroethylene (DDE) [35, 36]. The present study aimed to investigate the neurotoxic and apoptotic effects of BP-3 and the impact of this chemical on the expression and functions of ERs, including classical Erα/Erβ, the membrane Gpr30 and Pparγ. Neurotoxicity was estimated by measuring lactate dehydrogenase (LDH) release, which was complemented by an assessment of caspase-3 and -8 activities and a loss of mitochondrial membrane potential and ROS production. These data were supported by Hoechst 33342/calcein acetoxymethyl (AM) staining, which allowed for apoptotic nuclei and cell survival to be visualized, respectively, and by measurements of the expression of genes involved in apoptosis, as detected by a microarray analysis. Specific inhibitors of caspase-8, caspase-9, glycogen synthase kinase-3β (Gsk3β), and p38/MAPK were employed to indicate which of these enzymes participated in BP-3-induced caspase-3-dependent apoptosis. The involvement of classical and membrane ERs and Pparγ in BP-3 actions was verified using selective antagonists and agonists. The levels of specific messenger RNAs (mRNAs) and proteins were measured with quantitative polymerase chain reaction (qPCR), western blot, and ELISA, and the cellular distributions of receptors were demonstrated using a confocal microscope.

## Materials and Methods

### Materials

B27 and neurobasal media were obtained from Gibco (Grand Island, NY, USA). L-glutamine; fetal bovine serum (FBS); N-acetyl-Asp-Glu-Val-Asp *p*-nitro-anilide (Ac-DEVD-*p*NA); dimethyl sulfoxide (DMSO); HEPES; CHAPS; mouse monoclonal anti-MAP-2 antibody; ammonium persulfate; TEMED; TRIZMA base; Tween 20; DL-dithiothreitol; Nonidet NP-40; sodium deoxycholate; protease inhibitor (EDTA-free); bromophenol blue; 2′,7′-dichlorofluorescein diacetate; Gsk3β; p38/MAPK; caspase-9 and caspase-8 inhibitors (SB 216763, SB 203580, Z-Leu-Glu(O-Me)-His-Asp(O-Me) fluoromethyl ketone trifluoroacetate salt hydrate, Z-Leu-Glu(OMe)-Thr-Asp(OMe) fluoromethyl ketone); RIPA buffer; protease inhibitor cocktail for mammalian tissues; and poly-ornithine were obtained from Sigma-Aldrich (St. Louis, MO, USA). Bradford reagent, SDS, 30% acrylamide, 0.5 M Tris-HCl buffer, 1.5 M Tris-HCl gel buffer, and Laemmli sample buffer were from Bio-Rad Laboratories (Munchen, Germany). 4,4′,4″-(4-Propyl-[1H]-pyrazole-1,3,5-triyl)trisphenol (PPT), 2,3-bis(4-Hydroxyphenyl)-propionitrile (DPN), G1, ICI 182780, GW1929, methylpiperidino-pyrazole (MPP), 4-[2-phenyl-5,7-bis(trifluoromethyl)pyrazolo[1,5,-a]pyrimidin-3-yl]phenol (PHTPP), G15, and GW9662 were from Tocris Bioscience (Minneapolis, MN, USA). 2-mercaptoethanol was from Carl Roth GmbH+ Co. KG, (Karlsruhe, Germany). Immobilon-P membranes were purchased from Millipore (Bedford, MA, USA). Alexa 488-conjugated anti-goat IgG, calcein AM, and Hoechst 33342 were purchased from Molecular Probes (Eugene, OR, USA). Cy3-conjugated anti-rabbit IgG and Cy5-conjugated anti-mouse were obtained from Jackson ImmunoResearch, Inc. (West Grove, PA, USA). The cytotoxicity detection kit and BM Chemiluminescence Western Blotting Substrate (POD) were purchased from Roche Diagnostics GmbH (Mannheim, Germany). ELISA assay kits for Erα, Erβ, Gpr30 and Pparγ were purchased from Shanghai Sunred Biological Technology Co. (Sunred, China). The culture dishes were obtained from TPP Techno Plastic Products AG (Trasadingen, Switzerland). Rabbit polyclonal anti-Erα antibody (sc-7207), goat polyclonal anti-Erβ antibody (sc-6822), rabbit polyclonal anti-Erβ antibody (sc-8974), rabbit polyclonal anti-Gpr30 antibody (sc-134576), mouse monoclonal anti-Pparγ antibody (sc-7273), mouse monoclonal anti-β-Actin antibody (sc-47778), Erα siRNA (sc-29306), Erβ siRNA (sc-35326), Gpr30 siRNA (sc-60744), and Pparγ siRNA (sc-29456) were purchased from Santa Cruz Biotechnology, Inc. (Santa Cruz, CA, USA). AllStars Negative Control siRNA AF 488, RNeasy Mini Kit and RT^2^ Profiler PCR Apoptosis Array were obtained from QIAGEN (Valencia, CA, USA). INTERFERin was obtained from PolyPlus Transfection (Illkirch, France), and the high-capacity complementary DNA (cDNA)-Reverse Transcription Kit, the TaqMan Gene Expression Master Mix and TaqMan probes corresponding to specific genes encoding *Hprt*, *Erα*, *Erβ*, *Gpr30*, and *Pparγ* were obtained from Life Technologies Applied Biosystems (Foster City, CA, USA). JC-1 was obtained from Biotium, Inc. (Hayward, CA, USA).

### Primary Neocortical and Hippocampal Neuronal Cell Cultures

Neocortical and hippocampal tissues for primary cultures were prepared from Swiss mouse embryos (Charles River, Germany) at 15–17 days of gestation and cultured as previously described [37]. All procedures were performed in accordance with the National Institutes of Health Guidelines for the Care and Use of Laboratory Animals and approved by the Bioethics Commission in compliance with Polish Law (21 August 1997). Animal care followed official governmental guidelines, and all efforts were made to minimize suffering and the number of animals used. The cells were suspended in estrogen-free neurobasal medium with a B27 supplement on poly-ornithine (0.01 mg/ml)-coated multi-well plates at a density of 2.0 × 10^5^ cells/cm^2^. The cultures were maintained at 37 °C in a humidified atmosphere containing 5% CO_2_ for 7 days in vitro (DIV) prior to experimentation. The number of astrocytes, as determined by the content of intermediate filament glial fibrillary acidic protein (GFAP), did not exceed 10% for all cultures [38].

### Treatment

Primary neuronal cell cultures were exposed to BP-3 (1–100 μM) for 6 or 24 h. To assess whether the effects of BP-3 were tissue-dependent, we examined these effects in neocortical and hippocampal cultures. The involvement of ER signaling in BP-3-induced effects was verified with the high-affinity estrogen receptor antagonist ICI 182,780 (1 μM), also known to act as a membrane estrogen receptor Gpr30 agonist [39], the Erα antagonist methyl-piperidino-pyrazole (MPP; 1 μM), the Erα agonist 4,4′,4″-(4-Propyl-[1H]-pyrazole-1,3,5-triyl)trisphenol (PPT; 1 μM), the Erβ antagonist 4-[2-phenyl-5,7-bis(trifluoromethyl)pyrazolo[1,5,-a]pyrimidin-3-yl]phenol (PHTPP; 1 μM), the Erβ agonist 2,3-bis(4-Hydroxyphenyl)-propionitrile (DPN; 1 μM), the Gpr30 antagonist G-15 (10 μM), and the Gpr30 agonist G-1 (1 μM). BP-3-induced Pparγ activation was examined using the receptor agonist GW1929 (1 μM) and antagonist GW9662 (1 μM). For apoptotic signaling, we used a cell permeable Gsk3β inhibitor SB 216763 (3-(2,4-dichlorophenyl)-4-(1-methyl-1Hindol-3-yl)-1H-pyrrole-2,5-dione; 1 μM) and a p38/MAPK inhibitor SB 203580 (4-(4-fluorophenyl)-2-(4-methylsulfinylphenyl)-5-(4-pyridyl)-1H-imidazole; 1 μM) and caspase-8 and caspase-9 inhibitors: Z-Leu-Glu(O-Me)-Thr-Asp(O-Me)-fluoromethyl ketone (Z-LETD-FMK; 40 μM) and Z-Leu-Glu(O-Me)-His-Asp(O-Me)-fluoromethyl ketone trifluoroacetate salt hydrate (Z-LEHD-FMK; 40 μM), respectively. GW1929, GW9662, ICI 182780, MPP, PPT, DPN, and PHTPP were added to the culture media 45–60 min before BP-3 was added. The other agents were introduced simultaneously with BP-3. To avoid non-specific effects in our study, specific receptor ligands and SB 216763, SB 203580, and the caspase inhibitors were used at concentrations that did not affect the control levels of caspase-3 activity or LDH release. All the compounds were originally dissolved in DMSO and then further diluted in culture medium to maintain the DMSO concentration below 0.1%. The control cultures were treated with DMSO in concentrations equal to those used in the experimental groups.

### Identification of Apoptotic Cells

Apoptotic cells were detected via Hoechst 33342 staining at 24 h after the initial treatment, as previously described [37]. Neocortical cells cultured on glass coverslips were washed with 10-mM phosphate-buffered saline (PBS) and exposed to Hoechst 33342 (0.6 mg/ml) staining at room temperature (RT) for 5 min. The cells containing bright blue fragmented nuclei, indicating condensed chromatin, were identified as apoptotic cells. Qualitative analysis was performed using a fluorescence microscope (NIKON Eclipse 80i, NIKON Instruments Inc., Melville, NY, USA) equipped with a camera with BCAM Viewer© Basler AG software.

### Staining with Calcein AM

The intracellular esterase activity in the neocortical cultures was measured based on calcein AM staining at 24 h after the initial treatment with BP-3 [37]. To avoid the esterase activity present in the growth media, the cells were washed with PBS and incubated in 2 μM calcein AM in PBS at RT for 10 min. The cells displaying bright green cytoplasm were identified as living cells. Fluorescence intensity was monitored at Ex/Em 494/520 nm using a fluorescence microscope (NIKON Eclipse 80i, NIKON Instruments Inc., Melville, New York, USA) equipped with a camera with BCAM Viewer© Basler AG software.

### Assessment of Caspase-3 Activity

Caspase-3 activity was determined according to Nicholson, using samples treated for 6 or 24 h with BP-3 alone or in combination with the test compounds [40]. The assessment of caspase-3 activity was performed as previously described [38]. Cell lysates from neocortical and hippocampal cultures were incubated at 37 °C using a colorimetric substrate preferentially cleaved by caspase-3, called Ac-DEVD-*p*NA (N-acetyl-asp-glu-val-asp-*p*-nitro-anilide). The levels of *p*-nitroanilide were continuously monitored for 60 min using a Multimode Microplate Reader Infinite M200PRO (Tecan, Mannedorf, Switzerland). The data were analyzed using Magellan software, normalized to the absorbency of vehicle-treated cells and expressed as a percentage of control ±SEM from three to four independent experiments. The absorbance of blanks, acting as no-enzyme controls, was subtracted from each value.

### Measurement of Lactate Dehydrogenase Activity

To quantify cell death, lactate dehydrogenase (LDH) release from damaged cells into the cell culture media was measured 6 or 24 h after treatment with BP-3. LDH release was measured as previously described [41]. Cell-free supernatants from neocortical and hippocampal cultures were collected from each well and incubated at room temperature for 30 to 60 min with the appropriate reagent mixture according to the manufacturer’s instructions (Cytotoxicity Detection Kit) depending on the reaction progress. The intensity of the red color that formed in the assay, measured at a wavelength of 490 nm (Infinite M200pro microplate reader, Tecan Mannedorf, Switzerland), was proportional to both LDH activity and the number of damaged cells. The data were analyzed using Magellan software, normalized to the color intensity from vehicle-treated cells (100%) and expressed as a percentage of the control value from three to four independent experiments. The absorbance of blanks, acting as no-enzyme controls, was subtracted from each value.

### Assessment of Loss of the Mitochondrial Membrane Potential

The loss of the mitochondrial membrane potential was measured using a JC-1 Assay Kit, which utilizes the cationic dye 5,5,6,6-tetrachloro-1,1,3,3-tetraethylbenzimidazolylcarbo-cyanine iodide. In healthy cells, this dye aggregates and stains mitochondria bright red, whereas in apoptotic cells, the mitochondrial membrane potential collapses, and the dye remains in the cytoplasm in a green fluorescent monomeric form. The loss of mitochondrial membrane potential, a hallmark of apoptosis, was assessed in neocortical cultures 24 h after treatment with BP-3 (25 μM). The cells were incubated in the JC-1 solution for 25 min, and the red (550/600 nm) and green (485/535 nm) fluorescence intensities were measured using an Infinite M1000 microplate reader (Tecan, Austria). The data were analyzed using Tecan i-control software, normalized to the fluorescence intensity of vehicle-treated cells, and expressed as the red to green fluorescence ratio ±SEM of three to four independent experiments. The fluorescence intensity of blanks, acting as no-enzyme controls, was subtracted from each value.

### ROS Formation

To determine the ability of BP-3 to induce ROS production in the neocortical neurons, 5 μM H2DCFDA was applied as previously described [42, 43]. After diffusion into the cell, H2DCFDA is deacetylated by cellular esterases into a non-fluorescent compound that is subsequently oxidized by ROS into 2,7′-dichlorofluorescein (DCF). The cells were incubated in serum- and phenol red-free neurobasal medium containing H2DCFDA for 40 min before BP-3. After this time, the culture medium was replaced with fresh medium to remove extracellular residual DCF. The DCF fluorescence was measured after 3–24 h of BP-3 treatment and detected using an Infinite M1000 microplate reader (Tecan, Austria). The data were analyzed using Tecan i-control software and normalized to the fluorescence intensity in vehicle-treated cells (% of control). The means ± SEM from eight separate samples were calculated from four independent experiments.

### Silencing of Erα, Erβ, Gpr30, and Pparγ

Specific siRNAs were used to inhibit Erα, Erβ, Gpr30, and Pparγ expression in neocortical cells. Each siRNA was applied separately for 6 h at 50 nM in antibiotic-free medium containing the siRNA transfection reagent INTERFERin™. After transfection, the culture media were changed, and the cells were incubated for 12 h before starting the experiment. Positive and negative siRNAs containing a scrambled sequence that did not lead to the specific degradation of any known cellular mRNA were used as controls. The effectiveness of mRNA silencing was verified through the measurement of specific mRNAs using qPCR.

### qPCR Analysis of mRNAs Specific to Genes Encoding the Receptors *Erα*, *Erβ*, *Gpr30*, and *Pparγ*

Total RNA was extracted from neocortical cells cultured for 7 DIV (approx. 1.5 × 10^6^ cells/sample) using an RNeasy Mini Kit (QIAGEN, Valencia, CA) according to the manufacturer’s instructions. The quantity of RNA was spectrophotometrically determined at 260 nm and 260/280 nm (ND/1000 UV/Vis; Thermo Fisher NanoDrop, USA). Two-step real-time PCR was performed. Both the reverse transcription reaction and quantitative polymerase chain reaction (qPCR) were run on a CFX96 Real-Time System (Bio-Rad, USA). The products of the reverse transcription reaction were amplified using TaqMan Gene Expression Master Mix containing TaqMan primer probes specific to the genes encoding *Hprt*, *Erα*, *Erβ*, *Gpr30*, and *Pparγ.* Amplification was performed in a total volume of 20 μl of a mixture containing 10 μl TaqMan Gene Expression Master Mix and 1.0 μl reverse transcription product as the PCR template. A standard qPCR procedure was performed: 2 min at 50 °C and 10 min at 95 °C followed by 40 cycles of 15 s at 95 °C and 1 min at 60 °C. The threshold value (Ct) for each sample was set during the exponential phase, and the delta delta Ct method was used for data analysis. *Hprt* (hypoxanthine phosphoribosyltransferase coding gene) was used as a reference gene.

### Mouse Apoptosis RT^2^ Profiler PCR Array

Total RNA was extracted from neocortical cells cultured for 7 DIV (approx. 1.5 × 10^6^ cells/sample) using the RNeasy Mini Kit (QIAGEN, Valencia, CA) according to the manufacturer’s instructions. The quantity of RNA was spectrophotometrically determined at 260 and 260/280 nm (ND/1000 UV/Vis; Thermo Fisher NanoDrop, USA). A total of 1 μg mRNA was reverse transcribed to cDNA using an RT^2^ First Strand Kit (QIAGEN, Valencia, CA) and suspended in a final solution of 20 μl. Each cDNA was prepared for further use in qPCR. To analyze the signaling pathway, the RT^2^ Profiler™ PCR Array System (QIAGEN, Valencia, CA) was used according to the manufacturer’s protocol. The C_t_ values for all wells were exported to a blank Excel spreadsheet and were used with the Web-based software (www.SABiosciences.com/pcrarraydataanalysis.php).

### Western Blot Analysis

The cells exposed for 24 h to BP-3 were lysed in ice-cold RIPA lysis buffer containing a protease inhibitor cocktail. The lysates were sonicated and centrifuged at 15,000×g for 20 min at 4 °C. The protein concentrations in the supernatants were determined using Bradford reagent (Bio-Rad Protein Assay) with bovine serum albumin (BSA) as the standard. Samples containing 40 μg of total protein were reconstituted in the appropriate amount of sample buffer comprising 125 mM Tris, pH 6.8, 4% SDS, 25% glycerol, 4 mM EDTA, 20 mM DTT, and 0.01% bromophenol blue, denatured and separated on 7.5% SDS-polyacrylamide gel using a Bio-Rad Mini-Protean II Electrophoresis Cell, as previously described [44]. After electrophoretic separation, the proteins were electrotransferred to PVDF membranes (Millipore, Bedford, MA, USA) using the Bio-Rad Mini Trans-Blot apparatus. Following the transfer, the membranes were washed, and non-specific binding sites were blocked with 5% dried milk and 0.2% Tween-20 in 0.02 M Tris-buffered saline (TBS) for 2 h while shaking. The membranes were incubated overnight (at 4 °C) with one of the following primary antibodies (Santa Cruz Biotechnology): anti-Erα rabbit polyclonal antibody (diluted 1:200), anti-Erβ rabbit polyclonal antibody (diluted 1:200), anti-Gpr30 rabbit polyclonal antibody (diluted 1:150), anti-Pparγ mouse polyclonal antibody (diluted 1:150) or anti-β-actin mouse monoclonal antibody (diluted 1:3000) diluted in TBS/Tween. The signals were developed by chemiluminescence (ECL) using BM Chemiluminescence Blotting Substrate (Roche Diagnostics GmBH) and visualized using a Luminescent Image Analyzer Fuji-Las 4000 (Fuji, Japan). Immunoreactive bands were quantified using a MultiGauge V3.0 image analyzer.

### Enzyme-Linked Immunosorbent Assays for Erα, Erβ, Gpr30, and Pparγ

The levels of Erα, Erβ, Gpr30, and Pparγ were determined in neocortical cells 24 h after treatment with BP-3. Specific detection of these proteins was obtained using enzyme-linked immunosorbent assays (ELISAs) and the quantitative sandwich enzyme immunoassay technique. A 96-well plate was pre-coated with monoclonal antibodies specific to Erα, Erβ, Gpr30, or Pparγ. The standards and non-denatured cell extracts were added to the wells with biotin-conjugated polyclonal antibodies specific for Erα, Erβ, Gpr30, or Pparγ. Therefore, all native Erα, Erβ, Gpr30, or Pparγ proteins were captured using the immobilized antibodies. The plates were washed to remove any unbound substances, and horseradish peroxidase-conjugated avidin was added to interact with the biotin bound to Erα, Erβ, Gpr30, or Pparγ. After washing, the substrate solution was added to the wells. The enzyme reaction yielded a blue product. The absorbance was measured at 450 nm and was proportional to the amount of Erα, Erβ, Gpr30, or Pparγ. The protein concentration was determined in each sample using Bradford reagent (Bio-Rad Protein Assay).

### Immunofluorescent Labeling of Erα, Erβ, Gpr30, and Pparγ and Confocal Microscopy

For immunofluorescence detection of Erα, Erβ, Gpr30, and Pparγ, neocortical cells were grown on glass coverslips and subjected to immunofluorescence double-labeling, as previously described [45]. After 1 h of incubation in a blocking buffer (5% normal donkey serum and 0.3% Triton X-100 in 0.01 M PBS), the cells were treated for 24 h (at 4 °C) using five primary antibodies: rabbit polyclonal anti-Erα antibody (1:50), goat polyclonal anti-Erβ antibody (1:50), rabbit polyclonal anti-Gpr30 antibody (1:50), mouse monoclonal anti-Pparγ antibody (1:50), and anti-MAP2 mouse monoclonal (1:100), followed by a 24-h incubation in a mixture of secondary antibodies, including Alexa Fluor 488-conjugated anti-rabbit IgG (1:300), Cy3-conjugated anti-rabbit IgG (1:300), and Cy5-conjugated anti-mouse IgG (1:300). The samples were subsequently washed, mounted, coverslipped and analyzed using an LSM510 META, Axiovert 200 M confocal laser scanning microscope (Carl Zeiss MicroImaging GmbH, Jena, Germany) under a Plan-Neofluar 40×/1.3 Oil DIC objective. An He/Ne laser and an argon laser, with two laser lines emitting at 488, 514, and 633 nm, were used to excite the Alexa Fluor 488-, Cy3- and Cy5-conjugated antibodies, respectively. The fluorescence signal was enhanced after summing four scans per line. A pinhole value of 1 airy unit was used to obtain flat images.

### Data Analysis

Statistical tests were performed on raw data expressed as the mean arbitrary absorbance or fluorescence units per well containing 50,000 cells (measurements of caspase-3, LDH), the fluorescence units per 1.5 million cells (qPCR), the mean optical density per 40 μg of protein (Western blotting) or picogram of Erα, Erβ, Gpr30, and Pparγ per microgram of total protein (ELISAs). One-way analysis of variance (ANOVA) was preceded by the Levene test of homogeneity of variances and used to determine overall significance. Differences between control and experimental groups were assessed using a post hoc Newman–Keuls test, and significant differences were designated as *p < 0.05, **p < 0.01, ***p < 0.001 (versus control cultures), ^#^ *p* < 0.05, ^##^ *p* < 0.01, and ^###^ *p* < 0.001 (versus the cultures exposed to BP-3) and ^$^ *p* < 0.05 and ^$$$^ *p* < 0.001 (versus the siRNA-transfected control cultures). The results were expressed as the mean ± SEM of three to four independent experiments. The number of replicates in each experiment ranged from 2 to 3, except for measurements of caspase-3 activity and LDH release, with replicates ranging from 5 to 8. To compare the effects of BP-3 in various brain tissues and different treatment paradigms, the results corresponding to caspase-3, LDH, and western blot analysis were presented as a percentage of the control.

## Results

### Effects of BP-3 on Caspase-3 and Caspase-8 Activities and LDH Release and Mitochondrial Membrane Potential in 7 DIV Neocortical Cultures

**Fig. 1:**
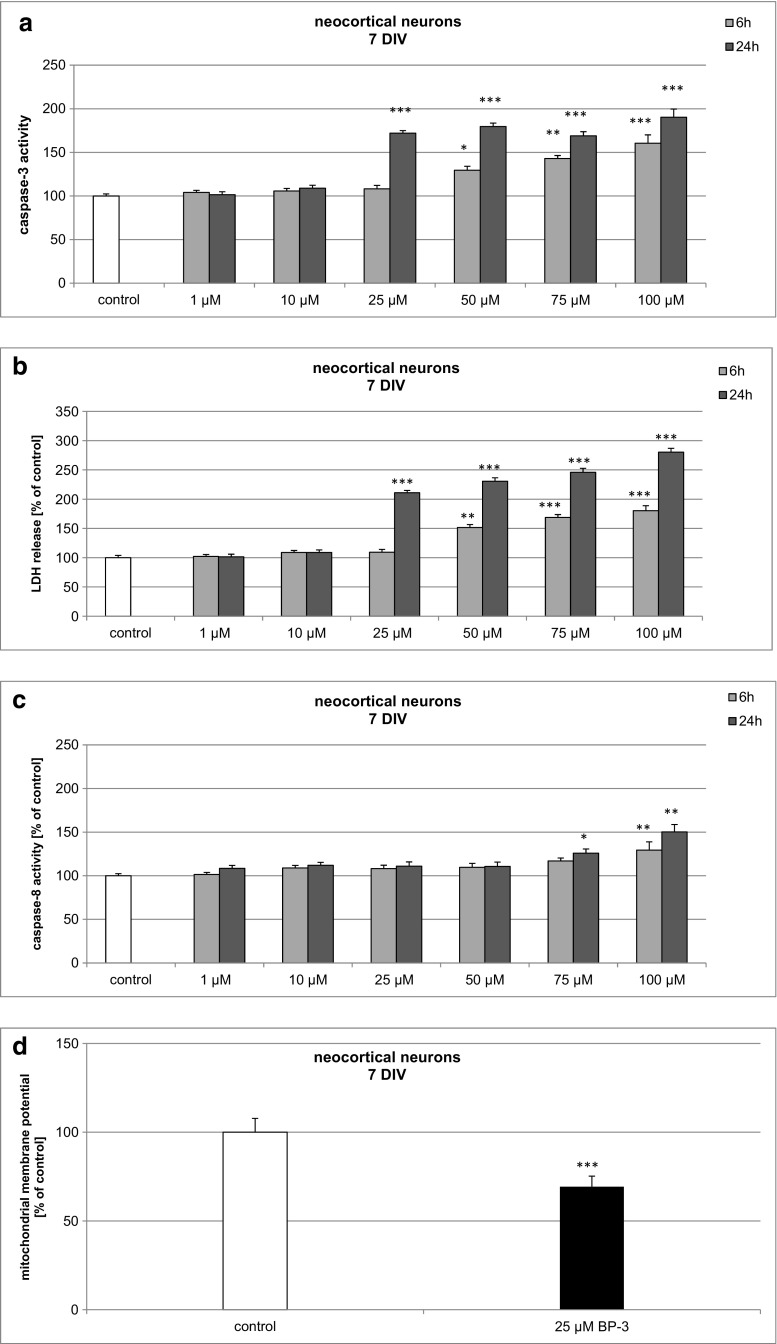
Time-course effects of BP-3 (1, 10, 25, 50, 75, and 100 μM) on caspase-3 activity (**a**), LDH release (**b**), caspase-8 activity (**c**), and mitochondrial membrane potential (**d**) in primary cultures of mouse neocortical cells at 7 DIV. The cells were treated with BP-3 for 6 and 24 h. The results are presented as a percentage of the control. Each *bar* represents the mean of three to four independent experiments ± SEM. The number of replicates in each experiment ranged from 5 to 8. *p < 0.05, **p < 0.01, and ***p < 0.001 versus control cultures

In 7 DIV neocortical cultures, BP-3 (25–100 μM)-induced caspase-3 increased to 160% of the control level at 6 h and was further enhanced to 190% at 24 h post-treatment (Fig. 1a). In these cells, LDH release values increased in neocortical cells in a time-dependent manner to 150–180% of the control value at 6 h and to 210–280% at 24 h (Fig. 1b). BP-3 at concentrations of 1–50 μM was not effective at activating caspase-8, but BP-3 at concentrations of 75–100 μM increased enzyme activity to 125–150% of the control value. (Fig. 1c). In 7 DIV cultures, treatment with 25 μM BP-3 decreased the mitochondrial membrane potential by 31% (Fig. 1d).

### Effects of BP-3 on Caspase-3 Activity and LDH Release in 7 DIV Hippocampal Cultures

**Fig. 2:**
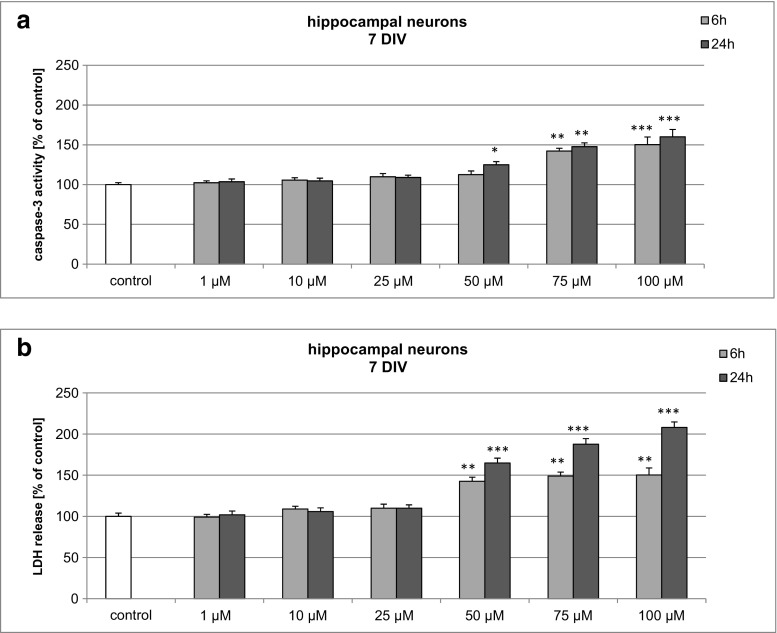
Time-course effects of BP-3 (1, 10, 25, 50, 75, and 100 μM) on caspase-3 activity (**a**) and LDH release (**b**) in primary cultures of mouse hippocampal cells at 7 DIV. The cells were treated with BP-3 for 6 and 24 h. The results are presented as a percentage of the control. Each *bar* represents the mean of three to four independent experiments ± SEM. The number of replicates in each experiment ranged from 5 to 8. *p < 0.05, **p < 0.01, and ***p < 0.001 versus control cultures

In hippocampal cultures exposed to 1–100 μM BP-3, the activity of caspase-3 increased to 150% at 6 h and to 160% at 24 h (Fig. 2a). LDH release increased with the duration of BP-3 treatment and was elevated to 150 and 208% of the vehicle control at 6 and 24 h, respectively (Fig. 2b). BP-3-induced caspase-3 activity and LDH release in hippocampal cells were lower than those in neocortical cells.

### Effects of BP-3 on Caspase-3 Activity and LDH Release in 2 and 12 DIV Primary Cultures of Mouse Neocortical Cells

**Fig. 3:**
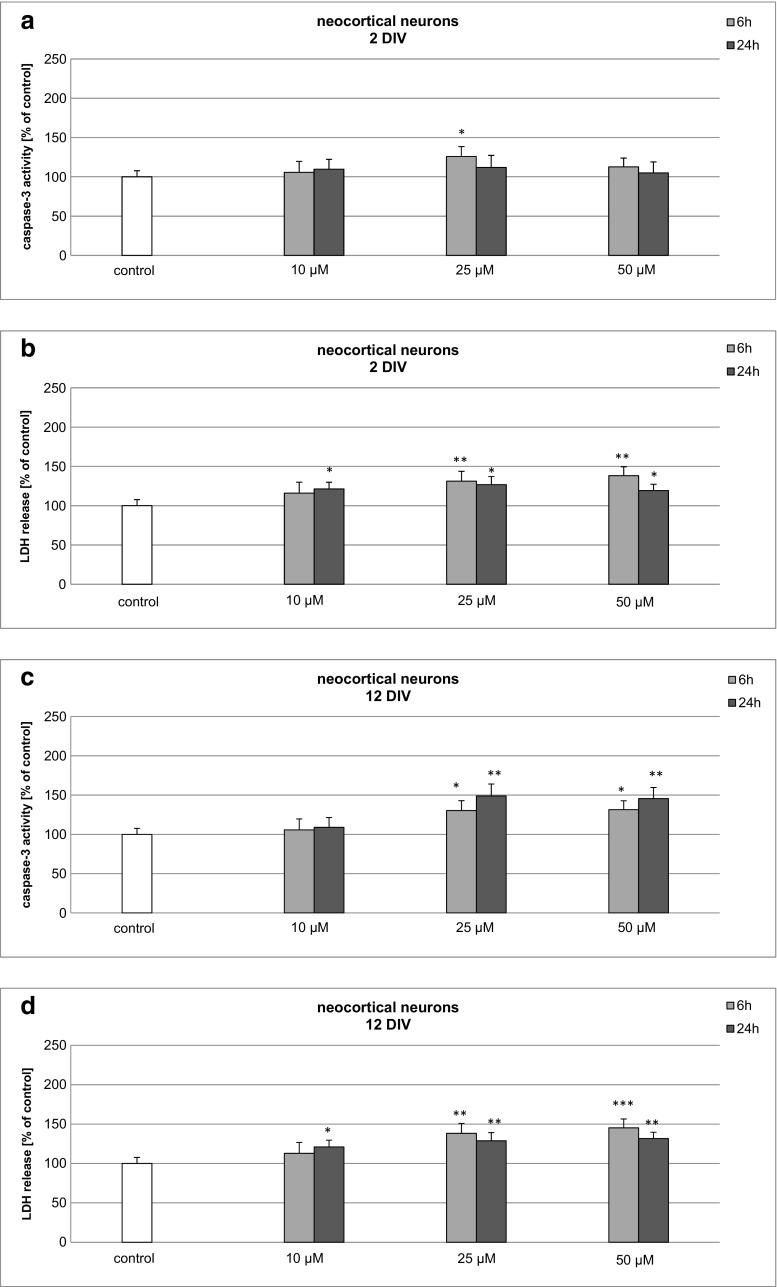
Time-course effects of BP-3 (10, 25, 50 μM) on caspase-3 activity (**a**, **c**) and LDH release (**b**, **d**) in primary cultures of mouse neocortical cells at 2 and 12 DIV. The cells were treated with BP-3 for 6 and 24 h. The results are presented as a percentage of the control. Each *bar* represents the mean of three to four independent experiments ± SEM. The number of replicates in each experiment ranged from 5 to 8. *p < 0.05, **p < 0.01, and ***p < 0.001 versus control cultures

To assess whether the effects of BP-3 depended on the age of the neuronal tissue, we studied them in 2 and 12 DIV neocortical cultures. In 2 DIV cultures, 25 μM BP-3 activated caspase-3 to 120% of the control value (Fig. 3a). BP-3-induced LDH release was enhanced to 131–138% of the control level at 6 h, after which it decreased to 119–126% at 24 h (Fig. 3b).

In 12 DIV cultures treated with 25–50 μM BP-3, the activity of caspase-3 rose to 131 and 148% of the control level at 6 and 24 h, respectively (Fig. 3c). In these cells, the LDH release was elevated to 121–145% of the control value (Fig. 3d). The effects of BP-3 in 2 and 12 DIV neocortical cultures were lower compared to the effects in 7 DIV neocortical cultures.

### Effects of BP-3 on ROS Production in 7 DIV Primary Cultures of Mouse Neocortical Cells

**Fig. 4:**
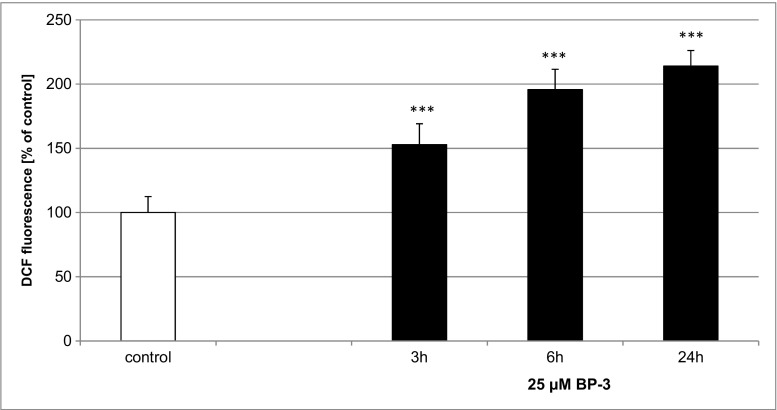
The effect of 25 μM BP-3 on ROS production after 3, 6, and 24 h. The data are expressed as the means ± SEM of four independent experiments, each of which consisted of eight replicates per treatment group. ***p < 0.001 versus the control

Following exposure to 25 μM BP-3, ROS production increased in the neurons in a time-dependent manner. Compared with the controls, ROS production reached values of 153% at 3 h, 196% at 6 h, and 214% at 24 h (Fig. 4).

### Impact of the Caspase-9, Caspase-8, Gsk3β, and p38/MAPK Inhibitors on BP-3-Induced Caspase-3 Activity and LDH Release

**Fig. 5:**
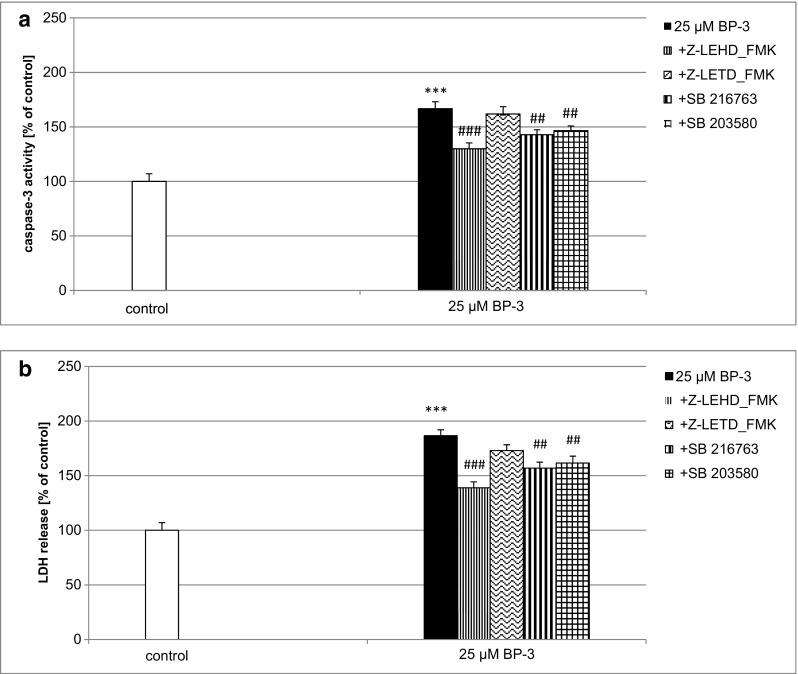
Impact of the caspase-9, caspase-8, Gsk3β, and p38/MAPK inhibitors on BP-3-induced caspase-3 activity and LDH release in 7 DIV neocortical cultures. Primary neocortical cultures were treated with BP-3 (25 μM) for 24 h. The caspase-9 inhibitor Z-LEHD-FMK (40 μM), caspase-8 inhibitor Z-LETD-FMK (40 μM), Gsk3β inhibitor SB 216763 (1 μM) and p38/MAPK inhibitor SB 203580 (1 μM) were added into the culture media simultaneously with BP-3. The results were normalized to the absorbency in vehicle-treated cells and expressed as a percentage of the control. Each *bar* represents the mean of three to four independent experiments ± SEM. The number of replicates in each experiment ranged from 5 to 8. ***p < 0.001 versus control cultures; ^##^ *p* < 0.01, and ^###^ *p* < 0.001 versus the cultures exposed to BP-3

In the presence of the caspase-9 inhibitor Z-LEHD-FMK (40 μM), the activity of caspase-3 was diminished by 78% at 24 h of exposure (Fig. 5a). A 24-h treatment with Gsk3β inhibitor SB 216763 (1 μM) and p38/MAPK inhibitor SB 203580 (1 μM) decreased BP-3-stimulated caspase-3 activity by 55 and 31%, respectively (Fig. 5a). However, exposure to the caspase-8 inhibitor Z-LETD-FMK (40 μM) did not significantly affect the BP-3-activated caspase-3 in mouse neocortical cells at 7 DIV.

In neocortical cultures, caspase-9, Gsk3β, and p38/MAPK inhibitors decreased BP-3-stimulated LDH release by 67, 57, and 46%, respectively (Fig. 5b). Co-treatment with caspase-8 inhibitor Z-LETD-FMK (40 μM) did not significantly influence the BP-3-induced effect in 7 DIV neocortical cultures.

### Effects of BP-3 on the Expression Profiles of Genes Involved in Apoptosis Using a Mouse Apoptosis RT^2^ Profiler PCR Array.

**Fig. 6:**
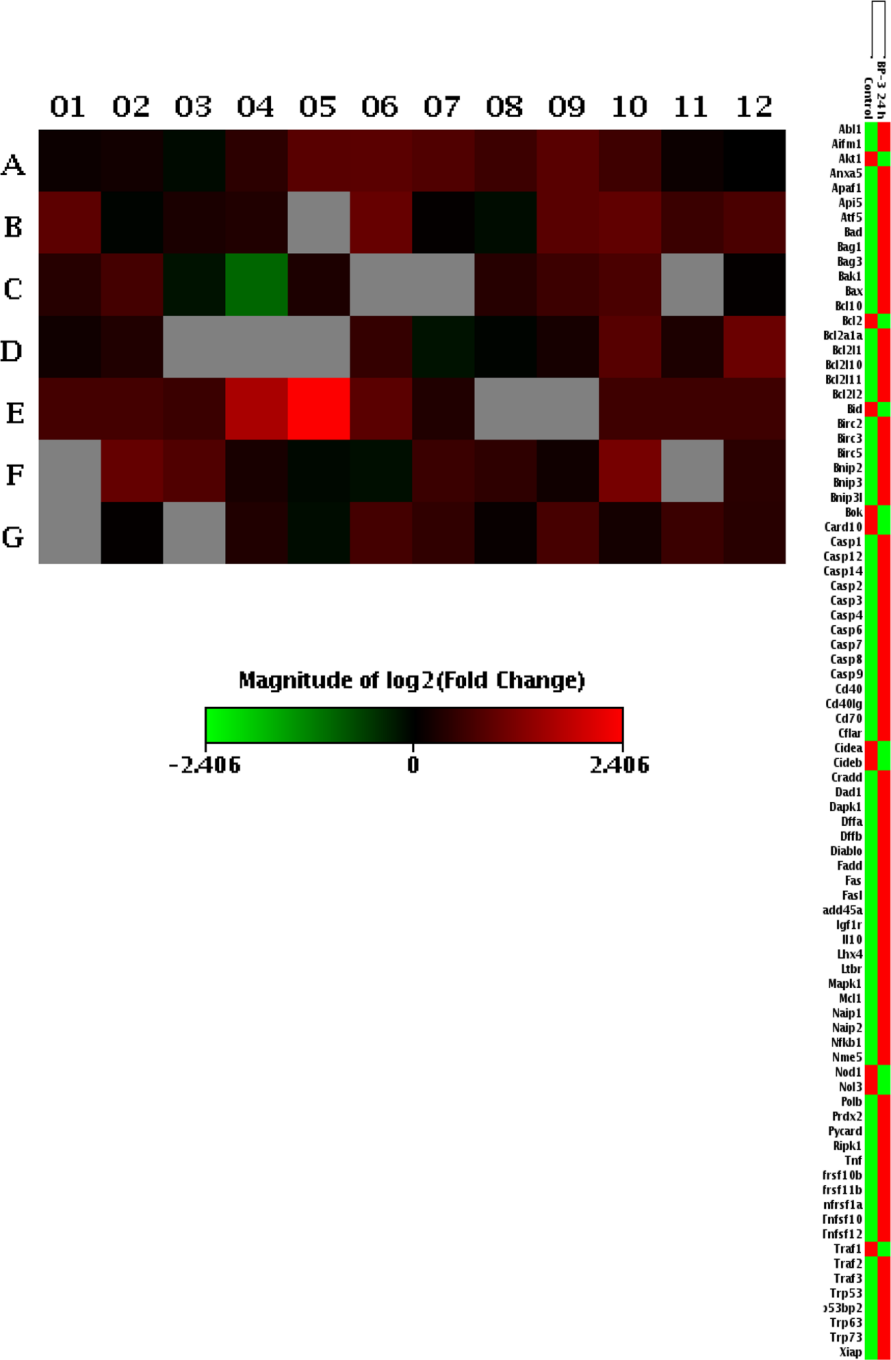
Gene expression patterns of apoptosis in the control and BP-3 groups showing the 46 genes that were differentially expressed with statistical significance between control and BP-3 group. Among these genes, 45 genes were upregulated (*red color*), and 1 gene was downregulated (*green panel*) in the BP-3-treated samples

To validate that BP-3 induced apoptosis in neuronal cells, we analyzed a total number of 84 key genes involved in programmed cell death. Among them, 46 genes were differentially expressed in response to BP-3: 45 were upregulated (red color) and 1 was downregulated (green color) in the BP-3-treated samples. The upregulated genes were *Anxa5*, *Apaf1*, *Api5*, *Atf5*, *Bad*, *Bag1*, *Bag3*, *Bcl10*, *Bcl2l11*, *Birc2*, *Birc3*, *Birc5*, *Bnip2*, *Bnip3*, *Bnip3l*, *Card10*, *Casp1*, *Casp2*, *Casp3*, *Casp4*, *Casp9*, *Cflar*, *Dad1*, *Dffa*, *Dffb*, *Diablo*, *Fadd*, *Fas*, *Fasl*, *Gadd45a*, *Igf1r*, *Ltbr*, *Mapk1*, *Mcl1*, *Naip2*, *Nfkb1*, *Polb*, *Prdx2*, *Ripk1*, *Tnfrsf10b*, *Tnfsf12*, *Traf2*, *Traf3*, *Trp53bp2*, *Trp73*, and *Xiap*. The only downregulated gene was *Card10* (Fig. 6).

### Effect of BP-3 on mRNA Levels of *Erα*, *Erβ*, *Gpr30*, and *Pparγ*

**Fig. 7:**
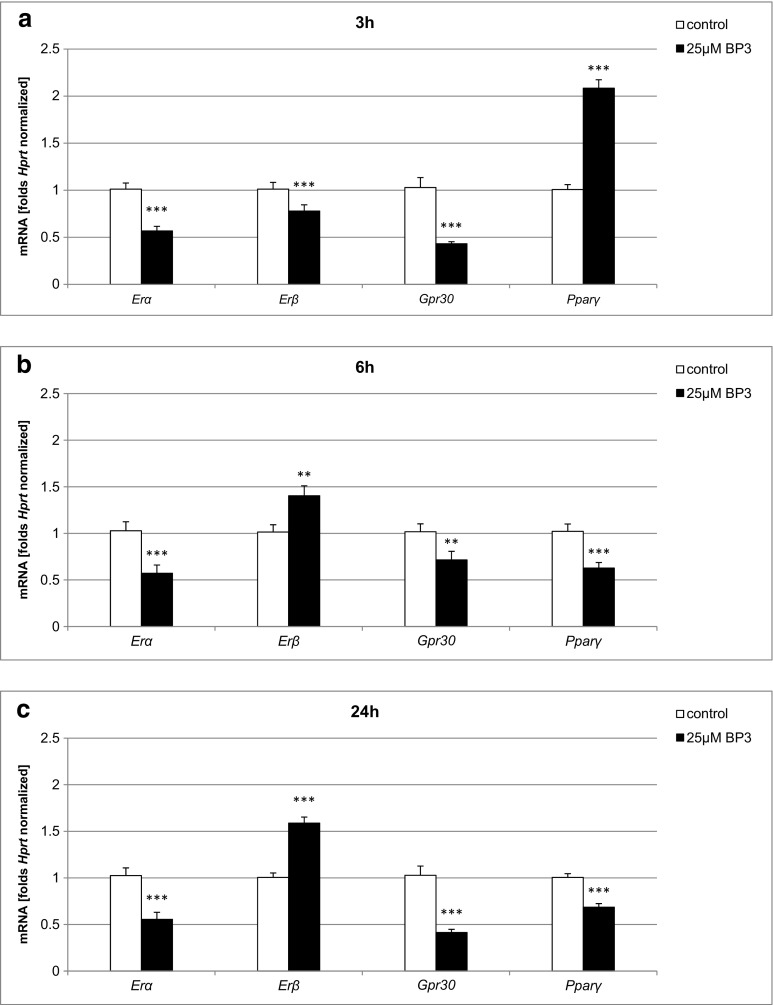
Effect of BP-3 (25 μM) on the mRNA expression levels of *Erα*, *Erβ*, *Gpr30*, and *Pparγ* in neocortical cultures at 7 DIV. The extraction of total RNA at 3, 6, and 24 h post-treatment from the neocortical cells was followed by reverse transcription and quantitative polymerase chain reaction (qPCR). The products of the reverse transcription reaction were amplified using TaqMan probes and primers corresponding to the specific genes. *Hprt* was used as a reference gene. Each *bar* represents the mean ± SEM of three independent experiments. The number of replicates for each experiment ranged from 2 to 3, **p < 0.01, and ***p < 0.001 versus control cultures

Treatment with BP-3 (25 μM) evoked changes in the mRNA levels of *Erα*, *Erβ*, *Gpr30*, and *Pparγ*. A 3-h exposure of the neocortical cultures to BP-3 caused a 45% decrease in *Erα*, a 23% decrease in *Erβ*, and a 60% decrease in *Gpr30* but caused a 100% increase in *Pparγ* mRNA compared with the control (Fig. 7a). The pattern of mRNA expression was changed after prolonged exposure to BP-3, especially with respect to *Erβ* and *Pparγ*. At 6 and 24 h of the experiment, BP-3 stimulated the mRNA expression of *Erβ* (39–58%) but inhibited the expression of *Pparγ* (32–39%) in neocortical cells (Fig. 7b, c). These data were normalized to *Hprt* as a control.

### Effects of BP-3 on the Protein Expression Levels of Erα, Erβ, Gpr30, and Pparγ in Mouse Neocortical Cells

**Fig. 8:**
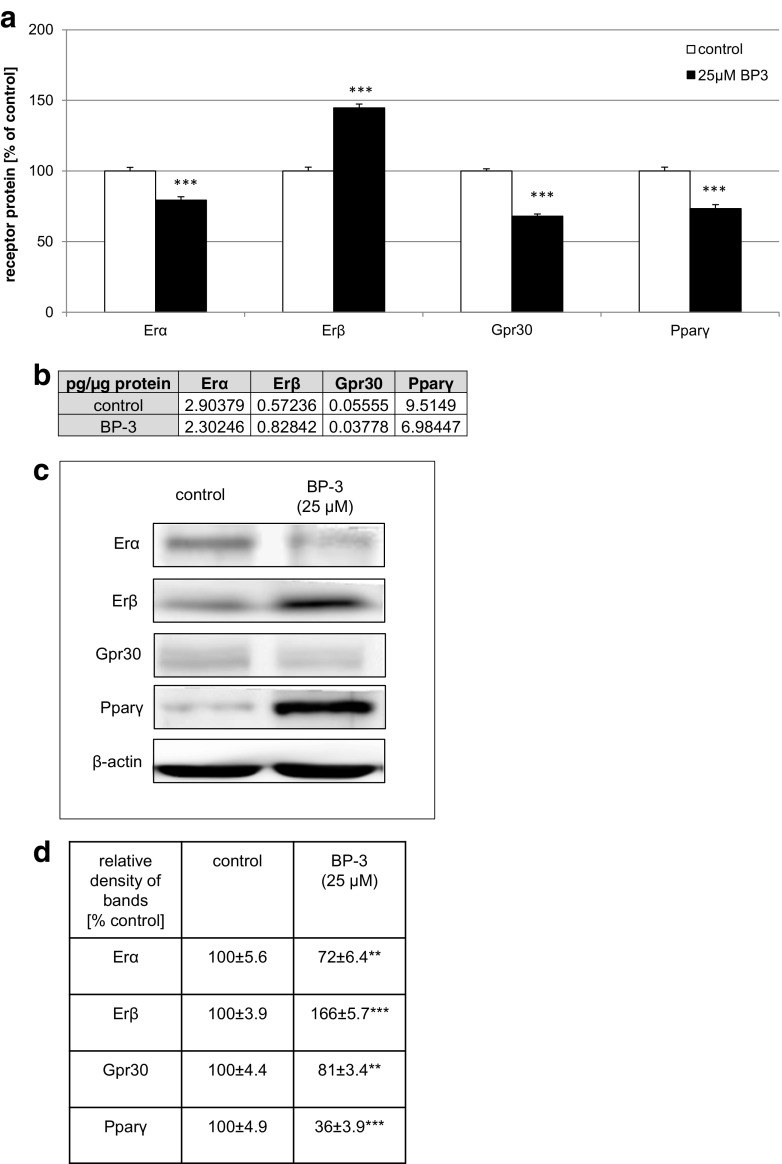
Effects of BP-3 on the protein levels of Erα, Erβ, Gpr30, and Pparγ in mouse neocortical cultures at 7 DIV. The neocortical cells were cultured for 7 DIV and then treated for 24 h with BP-3 (25 μM). The concentrations of the receptors were measured using specific ELISAs and are presented as a percentage of the control (**a**) and as pg of Erα, Erβ, Gpr30, and Pparγ per microgram of total protein (**b**). For the western blot analyses protein samples were denatured, electrophoretically separated, transferred to PVDF membranes, and subjected to immunolabeling (**c**). The signals were developed by chemiluminescence (ECL) and visualized using a Luminescent Image Analyzer Fuji-Las 4000 (Fuji, Japan). Immunoreactive bands were quantified using an image analyzer (ScienceLab, MultiGauge V3.0), and the relative protein levels of Erα, Erβ, Gpr30, and Pparγ were presented as a percentage of the control (**d**). Each *bar* or value represents the mean of three independent experiments ± SEM. The number of replicates in each experiment ranged from 2 to 3. **p < 0.01, and ***p < 0.001 versus control cultures

A 24-h exposure to BP-3 was necessary to detect changes in the protein levels of the receptors. In the control cultures, Erα and Pparγ reached 2.90 and 9.51 pg per microgram of total protein, respectively. A 24-h exposure to BP-3 (25 μM) decreased Erα and Pparγ levels by 21–27% of the control values (Fig. 8a, b).

In cultures exposed to BP-3 (25 μM) for 24 h, the concentration of Erβ was 0.83 pg per micrograms of total protein, and it was 45% larger than in control cultures (Fig. 8a, b). In the cultures exposed to BP-3, the level of Gpr30 reached 0.05 pg and it was reduced by 32% compared to the control.

The western blot analysis demonstrated the constitutive protein expression of Erα, Erβ, Gpr30, and Pparγ in mouse neocortical cells at 7 DIV (Fig. 8c). Exposure to BP-3 (25 μM) decreased the relative Erα, Gpr30, and Pparγ protein levels by 28, 19, and 64%, respectively, at 24 h post-treatment. Treatment with BP-3 (25 μM) increased the relative Erβ protein level by 66% (Fig. 8c, d).

### Effects of BP-3 Alone or in Combination with Receptor Agonists/Antagonists on BP-3-Induced Caspase-3 Activity and LDH Release in Neocortical Cultures

**Fig. 9:**
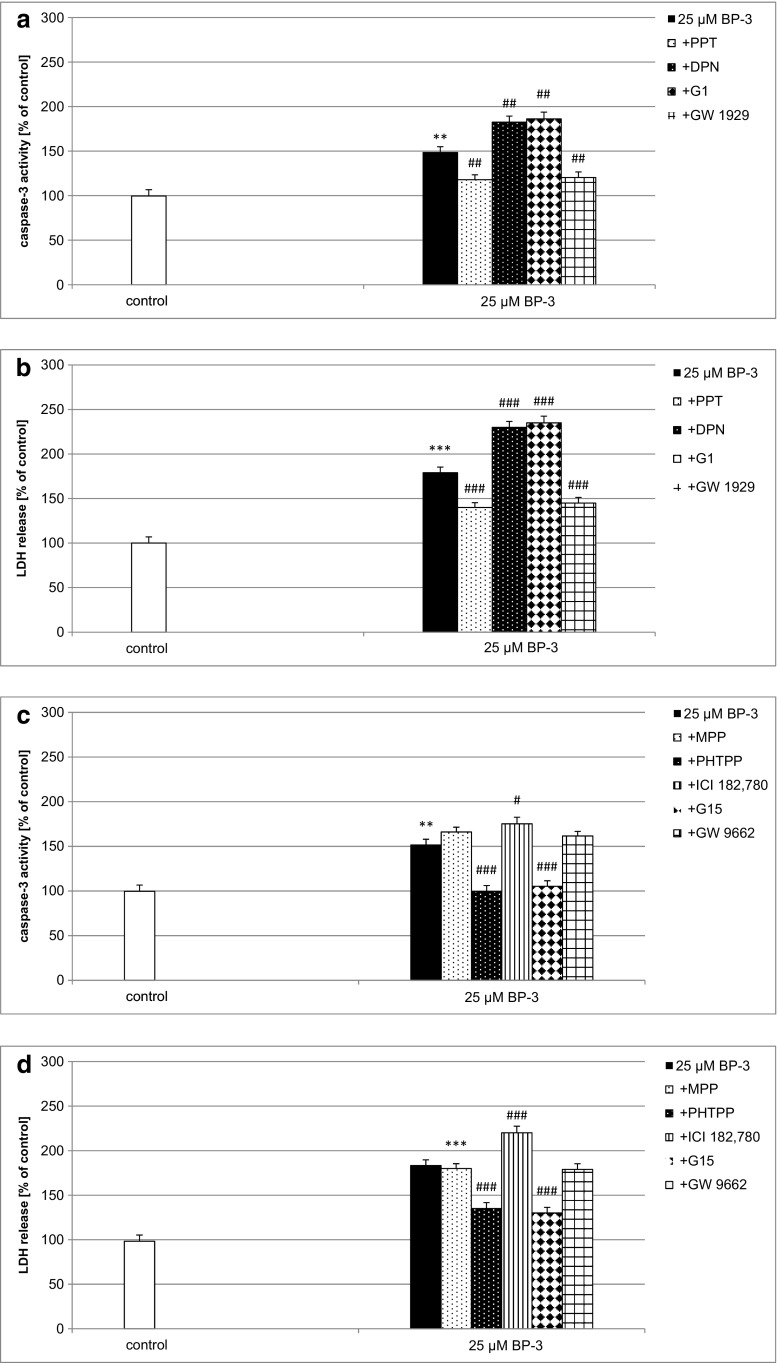
Impact of Erα, Erβ, Gpr30, and Pparγ agonists and antagonists on BP-3-induced caspase-3 activity (**a**, **c**) and LDH release (**b**, **d**) in 7 DIV neocortical cultures. The primary neocortical cultures were treated with BP-3 (25 μM) for 24 h. The Erα, Erβ, and Pparγ agonists (PPT, DPN, GW 1929) and antagonists (MPP, PHTPP, ICI 182780, GW9662; 1 μM) were added to the culture media approximately 45–60 min before BP-3 was added. The membrane ER agonist G1 (1 μM) and antagonist G15 (10 μM) were added to the culture media simultaneously with BP-3. The results were normalized to the absorbency in vehicle-treated cells and are expressed as a percentage of control. Each *bar* represents the mean of three to four independent experiments ± SEM. The number of replicates in each experiment ranged from 5 to 8. **p < 0.01, ***p < 0.001 versus control cultures, ^#^ *p* < 0.05, ^##^ *p* < 0.01 and ^###^ *p* < 0.001 versus the cultures exposed to BP-3

A 24-h exposure of neocortical cultures to BP-3 (25 μM) caused almost a 50% increase in caspase-3 activity in the neuronal cells. Co-treatment with the selective Erβ and Gpr30 agonists, DPN (1 μM) and G1 (10 μM), enhanced the action of BP-3 on caspase-3 activity by 21 and 56%, respectively. The selective Erα agonist PPT and Pparγ agonist GW 1929 (both 1 μM) significantly diminished the effect of 25 μM BP-3 in the mouse neuronal cell cultures (Fig. 9a).

The selective Erα agonist PPT (1 μM) and Pparγ agonist GW 1929 (1 μM) effectively inhibited BP-3-induced LDH release. In addition, high-affinity estrogen receptor antagonist ICI 182780 (1 μM) and selective Erβ or Gpr30 agonists intensified BP-3-induced LDH release (Fig. 9b).

The Erβ antagonist PHTPP (1 μM) and the Gpr30 antagonist G15 (10 μM) inhibited the BP-3 (25 μM)-induced caspase-3 activity by 52 and 46%, respectively, whereas the selective Erα antagonist MPP (1 μM) and the Pparγ antagonist GW 9662 did not change this effect. Treatment of the neocortical cultures with the high-affinity estrogen receptor antagonist ICI 182780 (1 μM), which was recently found to possess properties of a Gpr30 agonist, potentiated the BP-3-stimulated caspase-3 activity by 40% (Fig. 9c).

As demonstrated in Fig. 9d, 1 μM PHTPP or G15 reduced LDH release by 48 and 53% in the cultures subjected to BP-3 (25 μM) for 24 h. In this study, exposure to MPP or GW 9662 did not affect BP-3-induced LDH release in the mouse neocortical cultures. In addition, the high-affinity estrogen receptor antagonist ICI 182780 (1 μM) intensified BP-3-induced LDH release (Fig. 9d).

### Effects of BP-3 Alone or in Combination with MPP, PHTPP, G15, and GW 9662 on Hoechst 33342 and Calcein AM Staining in Neocortical Cultures

**Fig. 10:**
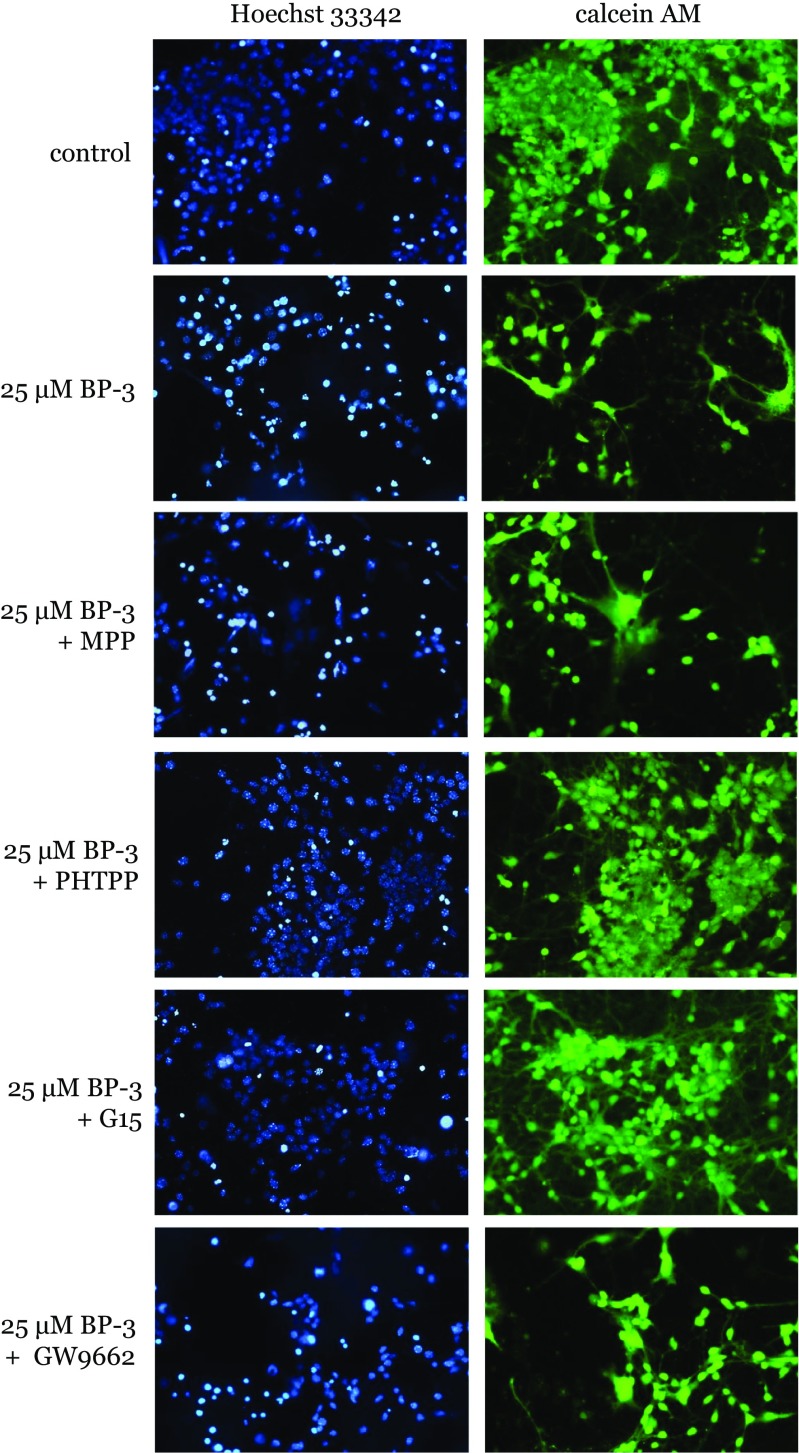
Influence of BP-3 (25 μM) and MPP (1 μM), PHTPP (1 μM), G15 (10 μM), GW 9662 (1 μM) on Hoechst 33,342 (first column) and calcein AM (second column) staining in mouse neocortical cultures at 7 DIV, examined 24 h post-treatment. The Erα, Erβ, and Pparγ antagonists (MPP, PHTPP, GW9662) were added to the culture media approximately 45–60 min before BP-3 was added. The membrane ER antagonist G15 (10 μM) was added to the culture media simultaneously with BP-3. The cells were cultured on glass coverslips, washed with 10 mM PBS, and exposed to Hoechst 33342 (0.6 μg/ml) at RT for 5 min. The cells were subsequently rewashed and incubated with 2 μM calcein AM at RT for 10 min. Cells with bright fragmented nuclei showing condensed chromatin were identified as undergoing apoptosis, whereas cells with light-colored cytoplasm were identified as living cells

In the present study, a 24-h exposure to BP-3 was necessary to develop an apoptotic morphology of cell nuclei. A continuous 24-h exposure of neocortical cultures to BP-3 (25 μM) induced apoptosis in mouse neuronal cells, as evidenced by Hoechst 33342 staining. The formation of bright blue fragmented nuclei containing condensed chromatin was labeled with Hoechst 33342 (Fig. 10). Treatment with BP-3 reduced the density of calcein AM-stained living cells at 7 DIV, as indicated by the decreased number of cells exhibiting light-colored cytoplasm. Co-treatment with MPP (1 μM) and GW 9662 (1 μM) did not significantly affect Hoechst 33342 and calcein AM staining. However, the addition of PHTPP (1 μM) and G15 (10 μM) inhibited the BP-3-induced effects.

### Influence of BP-3 on Caspase-3 Activity and LDH Release in Neocortical Cells Transfected with Erα, Erβ, Gpr30, and Pparγ siRNAs

**Fig. 11:**
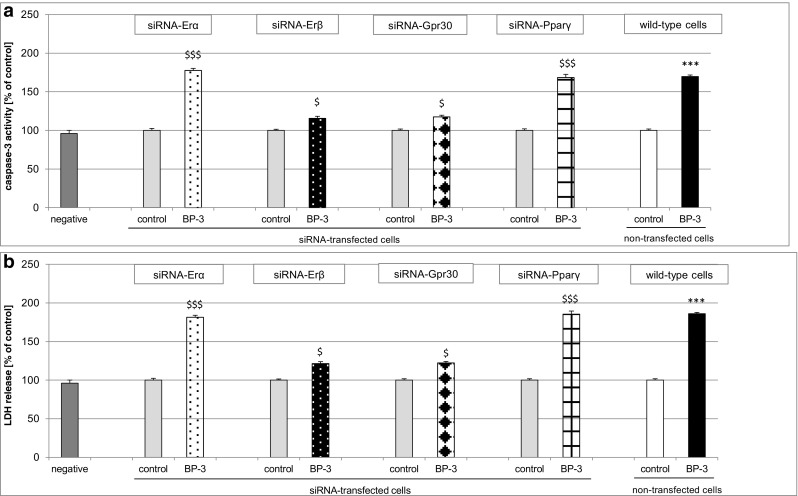
Effect of BP-3 (25 μM) on caspase-3 activity (**a**) and LDH release (**b**) in Erα, Erβ, Gpr30, and Pparγ siRNA-transfected neocortical cells. The primary neocortical cultures were transfected with 50 nM Erα, Erβ, Gpr30, and Pparγ siRNAs in INTERFERin™- containing medium without antibiotics for 6 h. The results were normalized to the absorbance in vehicle-treated cells and are expressed as a percentage of the control, either in siRNA-transfected or non-transfected cells. Each bar represents the mean ± SEM of three to four independent experiments. The number of replicates in each experiment ranged from 5 to 8. ***p < 0.001 versus the non-transfected control cultures, ^$^ *p* < 0.05, ^$$$^ *p* < 0.001 versus the siRNA-transfected control cultures

A 24-h exposure to BP-3 (25 μM) only slightly enhanced caspase-3 activity and LDH release in the Erβ and Gpr30 siRNA-transfected cells, suggesting that the transfected cells were less vulnerable to BP-3 than the non-transfected cells. In comparison to non-transfected cells, in the siRNA-transfected cells, the effects of BP-3 were reduced by 55% with respect to caspase-3 and by 65% with respect to LDH (Fig. 11a, b).

A 24-h exposure of Erα and Pparγ siRNA-transfected cells to 25 μM BP-3 increased caspase-3 activity and LDH release by 85 and 75% over the control, respectively (Fig. 11a, b). These cells were vulnerable to BP-3 in the same way as the non-siRNA-treated wild cells.

The effectiveness of mRNA silencing was verified by measuring the specific mRNAs using qPCR. In this study, mRNA silencing decreased the *Erα* mRNA concentration by 62% (equal to 0.38-fold), the *Erβ* mRNA concentration by 68% (equal to 0.32-fold), the Gpr30 mRNA concentration by 59% (equal to 0.41-fold), and the Pparγ mRNA concentration by 69% (equal to 0.31-fold) compared to the non-transfected wild-type cells.

### Effect of BP-3 on the Distribution of Erα, Erβ, Gpr30, Pparγ, and MAP2 Staining in Neocortical Cells.

**Fig. 12:**
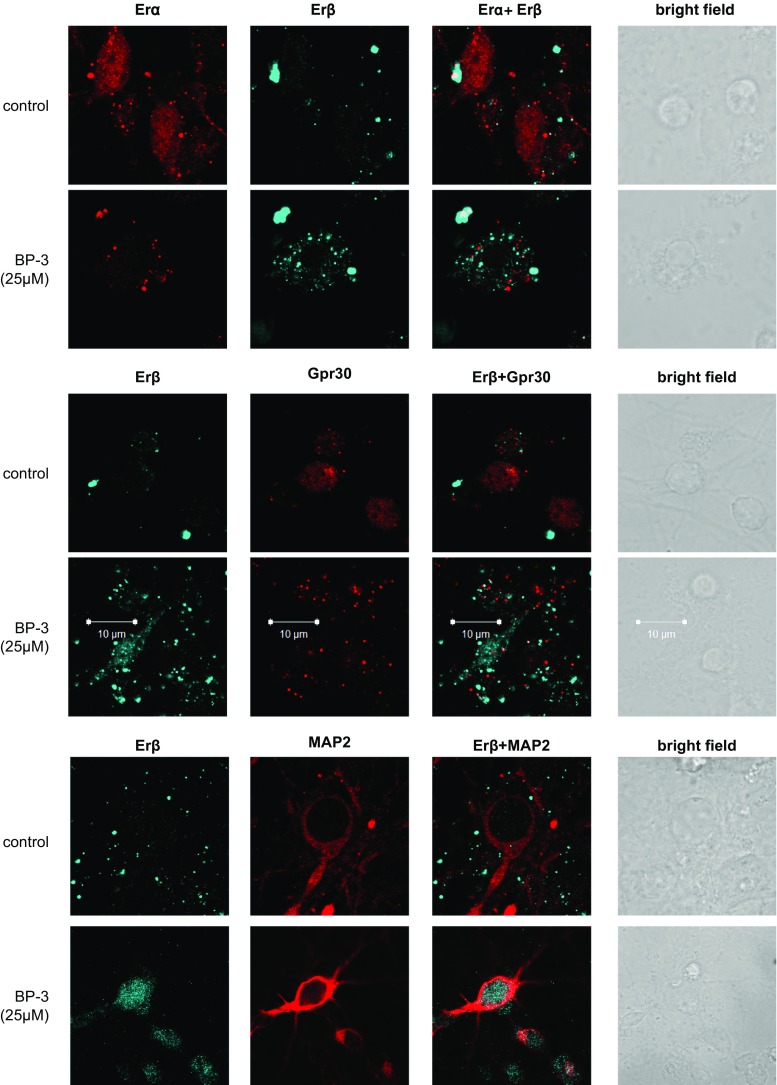

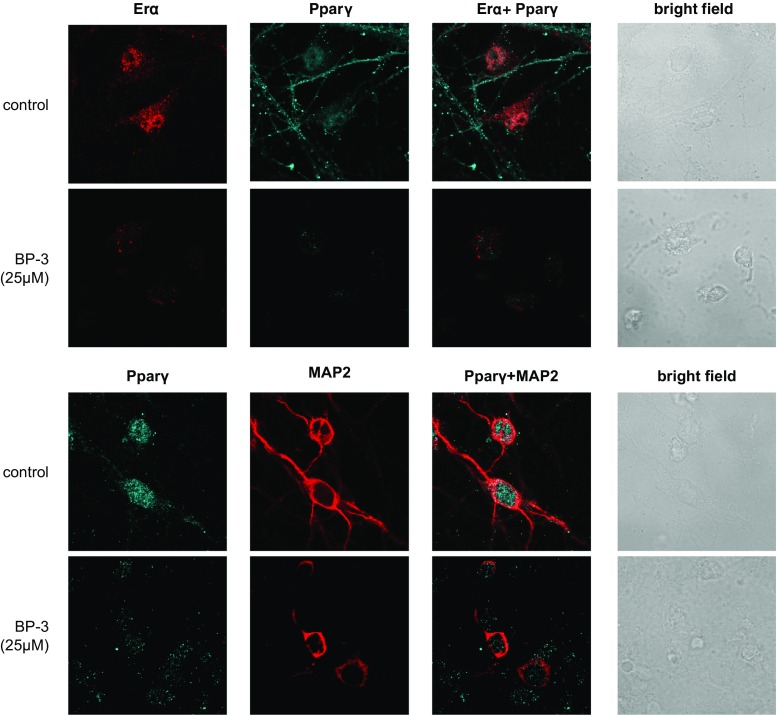
Influence of BP-3 on the cellular distributions of Erα (*red*), Erβ (*blue*), Gpr30 (*red*), Pparγ (*blue*), and MAP2 (*red*) in mouse neocortical cultures at 7 DIV. The overlay of Erα/Erβ, Erβ/Gpr30, Erα/Pparγ, Erβ/Map2, and Pparγ/MAP2 (*red plus blue*) and the bright field images are also shown. The primary neocortical cultures were treated with BP-3 (25 μM) for 24 h. The cells were cultured on glass coverslips and subjected to immunofluorescent double-labeling. The samples were analyzed using an LSM510 META Axiovert 200 M confocal laser scanning spectral microscope (Carl Zeiss MicroImaging GmbH, Jena, Germany) with a Plan-Apochromat 63×/1.4 Oil DIC objective

Immunofluorescence labeling and confocal microscopy revealed that Erα, Erβ, Gpr30, and Pparγ were localized to neocortical cells at 7 DIV. A 24-h exposure to BP-3 (25 μM) increased Erβ staining but reduced Erα-, Gpr30, and Pparγ-specific immunofluorescence. MAP2 staining confirmed the neural localization of receptors and revealed the BP-3-induced inhibition of neurite outgrowth (Fig. 12).

## Discussion

The primary aim of the present study was to evaluate the apoptotic and neurotoxic effects of BP-3 on neuronal cells with a particular emphasis on the molecular mechanisms of its actions, including estrogen receptors and Pparγ. We demonstrated that BP-3 evoked concentration-dependent activation of caspase-3 and LDH release in neocortical and hippocampal tissues. In the paradigms examined in the present study, neocortical tissue responded to 25–100 μM BP-3, whereas hippocampal cells exhibited weaker vulnerability. The extended vulnerability of neocortical cells to BP-3 could be due to low expression of ERs in this tissue which limits ER-related neuroprotection [46]. Indeed, in comparison to the hippocampal cells [42], in neocortical cells Erα protein is present in much lower concentration as evidenced in the present study. Recently, it has been observed that BP-3 used in similar concentrations (0.1–100 μM) caused toxic effects in neuroblastoma cells [20]. The BP-3 concentrations used in our study are environmentally relevant because BP-3 has been found in human adipose tissue at concentrations up to nearly 5 mg/kg, which is equal to approximately 22 μM [47]. In the cited article of Wang et al. [47], provided data on accumulation of BP-3 in adipose tissue were related to the concentrations of the compound in males and females of Caucasian and African-American origins and expressed as mean values. However, the authors underlined approximately threefold lower levels of accumulated BP-3 in African-Americans both males and females. Therefore, the actual levels of accumulated BP-3 in Caucasians could be much higher than the values of 30.3 and 62.2 ng/g. BP-3 is able to cross blood–brain barrier since it has altered Erα and Erβ mRNA expression levels in rat pituitary gland after being applied in gavages [13]. Moreover, BP-4 which is BP-3 analogue when added to water, displayed multiple effects on gene expression in the zebrafish brain [48].

In the present study, the biochemical alterations were accompanied by increased apoptotic body formation and impaired cell survival, evidenced by Hoechst 33342 and calcein AM staining and by the upregulation of genes involved in apoptosis detected by microarray analysis. The BP-3-induced effects were age-dependent with the most pronounced enzyme activities observed at 7, but not at 2 and 12 DIV. Based on our results, BP-3 induced ROS formation, suggesting that ROS initiated the intrinsic apoptotic pathway in neuronal cells. Indeed, we indicated that BP-3 caused a substantial loss of mitochondrial membrane potential, and inhibitors of intrinsic-related caspase-9 and kinase Gsk3β reduced the effects induced by BP-3. The cytotoxic effects of BP-3, which involved activation of caspase-3, LDH release, decreased mitochondrial membrane potential, and ROS formation, were previously observed in the HaCaT cell line [49]. Moreover, a reduction of ROS scavengers, i.e., GSH, in response to BP-3 was observed in *Tetrahymena thermophila* [50]. In our study, the apoptotic effects of BP-3 were inhibited by an inhibitor of p38/MAPK but not by an inhibitor of extrinsic-related caspase-8, thus confirming the prevalence of an intrinsic pathway in BP-3-induced apoptosis in neural cells. Furthermore, BP-3 only partially engaged extrinsic-related caspase-8 in neuronal apoptosis when used in higher concentrations. We postulate that BP-3 by stimulating p38/MAPK may inhibit Er an d Pparγ functions in neuronal cells.

Until now, UV filters, including BP-3, have been shown to have endocrine-disrupting capacities and act via sex steroid receptors, i.e., classical estrogen and androgen receptors. However, there is no data regarding the mechanisms of action of BP-3 in the mammalian nervous system that involve classical and newly recognized membrane estrogen receptors and Pparγ. Classical estrogen receptors (Erα, Erβ) and Pparγ are nuclear receptors acting as ligand-activated transcription factors. The estrogen receptors when located on the plasma membranes may rapidly activate intracellular pathways such as ERK 1/2 and PI3K kinases [51, 52]. As for Pparγ, there are no reports on membrane localization of the receptor. In the present study, BP-3 altered the mRNA expression levels of *Erα*, *Erβ*, *Gpr30*, and *Pparγ* in a time-dependent manner. We demonstrated that 3–24-h exposures caused decreases in *Erα* and *Gpr30* mRNAs. Moreover, 6–24-h exposures to BP-3 caused a significant increase in *Erβ* mRNA but decreased *Pparγ* mRNA. Recently, BP-3 was found to have a direct impact on the ER-related insect endocrine pathway, activating the ecdysone receptor gene *EcR* [53]. BP-3 also transcriptionally activated human *ERα* and *ERβ* in transfected human embryonic kidney cells HEK293 [54]. Furthermore, BP-3 was found to cause a strong activation of *hERβ* in reporter gene assays in HELN cells [55], which is similar to the substantial upregulation of *Erβ* mRNA observed in our study. We showed that BP-3-induced patterns of mRNA expression detected at 6 and 24 h reflected alterations in the protein levels of the receptors, as indicated by the use of specific ELISAs and western blot analyses. These profiles were also in parallel with the immunofluorescent labeling of Erα, Erβ, Gpr30, and Pparγ in response to BP-3. Basing on these data, we hypothesize that the BP-3-evoked apoptosis of neuronal cells is mediated via the attenuation of Erα/Gpr30/Pparγ and the stimulation of Erβ signaling pathways, though we did not differentiate the effects between nuclear and membrane receptors.

The proposed hypothesis has been partially confirmed by the use of selective receptor ligands and specific siRNAs. According to our data, Erα and Pparγ agonists diminished, but Erβ and Gpr30 agonists stimulated the BP-3-induced apoptotic and neurotoxic effects. In comparison to receptor agonists, receptor antagonists caused opposite effects, except for ICI 182,780, which is known to act not only as an Erα/Erβ antagonist but also as a Gpr30 agonist. These results were complemented by double Hoechst/calcein AM staining, according to which the receptor antagonists PHTPP and G15 improved viability and attenuated BP-3-induced apoptosis, whereas MPP and GW9662 did not change these parameters. This is in line with a substantial reduction of the effects of BP-3 in the cells with siRNA-silenced Erβ/Gpr30 and the maintenance of the effects of BP-3 in Erα- and Pparγ- siRNA-transfected cells. Therefore, we suggest that BP-3-evoked apoptosis of neuronal cells is mediated via an attenuation of Erα/Pparγ and stimulation of Erβ/Gpr30 signaling. Recently, it has been shown that activation of Pparγ inhibited caspase-3 activity and protected murine cortical neurons against ischemia [56, 57]. Reduced expression of Pparγ accompanied neurotoxicity of TBBPA [58] which is in line with the effect of BP-3 in our study. We hypothesize that the BP-3-evoked downregulation of Pparγ is at least partially due to the upregulation of Erβ. A negative cross-talk between Erβ and Pparγ has previously been shown in adipose tissue of βERKO mice [59]. It is also possible that the reduced expression of Erα observed in our study was linked to the cross-talk of Pparγ with its heterodimeric partner, the nuclear receptor Rxrα, through the mechanism previously detected in the vitellogenin A2 promoter [60]. We demonstrated that the BP-3-affected mRNA and protein expression levels of Erα, Erβ, and Pparγ measured by qPCR, ELISAs, western blot, and immunofluorescent labeling of neuronal cells are parallel to BP-3-induced apoptosis and neurotoxicity. Although BP-3 reduced Gpr30 mRNA and protein expression, it accelerated the function of Gpr30 in neuronal cells, thus pointing to the involvement of Gpr30 in BP-3 neurotoxicity.

## Conclusions

In summary, our study demonstrated that BP-3 caused neurotoxicity and activated apoptosis via an intrinsic pathway involving the loss of mitochondrial membrane potential and the activation of caspases-9 and -3 and kinases p38/MAPK and Gsk3β. We showed for the first time that BP-3-evoked apoptosis of neuronal cells is mediated via the attenuation of Erα/Pparγ and the stimulation of Erβ/Gpr30 signaling, as demonstrated using selective ligands and specific siRNAs and by measurements of mRNA and protein expression (qPCR, ELISA, western blot, immunofluorescent labeling) of the receptors.

AD, Alzheimer’s disease; ALS, amyotrophic lateral sclerosis; AM, acetoxymethyl; ANOVA, analysis of variance; BP-3, benzophenone-3; BSA, bovine serum albumin; CNS, central nervous system; DDE, dichlorodiphenyldichloroethylene; DDT, dichlorodiphenyltrichloroethane; DIV, days in vitro; DMSO, dimethyl sulfoxide; EDCs, endocrine-disrupting chemicals; ELISA, enzyme-linked immunosorbent assay; ERs, estrogen receptors; FBS, fetal bovine serum; GFAP, glial fibrillary acidic protein; Gpr30, G protein-coupled receptor 30; HD, Huntington’s disease; Hprt, hypoxanthine-guanine phosphoribosyltransferase; LDH, lactate dehydrogenase; mRNA, messenger RNA; PBS, phosphate-buffered saline; Ppar, peroxisome proliferator-activated receptors; qPCR, quantitative polymerase chain reaction; ROS, reactive oxygen species; RT, room temperature; Rxr, retinoid X receptor; SCI, spinal cord injuries; SEM, standard error of the mean; TBS, tris-buffered saline; UV, ultra violet light
